# Fast Procedures for the Electrodeposition of Platinum Nanostructures on Miniaturized Electrodes for Improved Ion Sensing

**DOI:** 10.3390/s19102260

**Published:** 2019-05-16

**Authors:** Francesca Criscuolo, Irene Taurino, Van Anh Dam, Francky Catthoor, Marcel Zevenbergen, Sandro Carrara, Giovanni De Micheli

**Affiliations:** 1 Laboratory of Integrated Systems, École Polytechnique Fédérale de Lausanne (EPFL), 1015 Lausanne, Switzerland; irene.taurino@gmail.com (I.T.); sandro.carrara@epfl.ch (S.C.); giovanni.demicheli@epfl.ch (G.D.M.); 2 Holst Centre, Interuniversity Microelectronics Centre (IMEC), 5656 AE Eindhoven, The Netherlands; Van.Anh.Dam@imec.nl (V.A.D.); Marcel.Zevenbergen@imec.nl (M.Z.); 3 Department ESAT, Interuniversity Microelectronics Centre (IMEC), 3001 Leuven, Belgium; Francky.Catthoor@imec.be

**Keywords:** platinum nanostructures, miniaturized electrodes, electrode nanostructuration, solid-contact, all-solid-state ion-selective electrode, potential drift

## Abstract

Nanostructured materials have attracted considerable interest over the last few decades to enhance sensing capabilities thanks to their unique properties and large surface area. In particular, noble metal nanostructures offer several advantages including high stability, non-toxicity and excellent electrochemical behaviour. However, in recent years the great expansion of point-of-care (POC) and wearable systems and the attempt to perform measurements in tiny spaces have also risen the need of increasing sensors miniaturization. Fast constant potential electrodeposition techniques have been proven to be an efficient way to obtain conformal platinum and gold nanostructured layers on macro-electrodes. However, this technique is not effective on micro-electrodes. In this paper, we investigate an alternative one-step deposition technique of platinum nanoflowers on micro-electrodes by linear sweep voltammetry (LSV). The effective deposition of platinum nanoflowers with similar properties to the ones deposited on macro-electrodes is confirmed by morphological analysis and by the similar roughness factor (~200) and capacitance (~18 μF/mm${}^{2}$). The electrochemical behaviour of the nanostructured layer is then tested in an solid-contact (SC) $Li^{+}$-selective micro-electrode and compared to the case of macro-electrodes. The sensor offers Nernstian calibration with same response time (~15 s) and a one-order of magnitude smaller limit of detection (LOD) ($2.6\times 10^{-6}$) with respect to the macro-ion-selective sensors (ISE). Finally, sensor reversibility and stability in both wet and dry conditions is proven.

## 1. Introduction

Over the last decade, nanostructured materials have been demonstrated to have exceptional properties in the improvement of sensing performance. Thanks to their unique behaviour and increased surface area, they can represent an effective mean to enhance sensor detection capabilities in different ways. They can be exploited to lower the limit of detection (LOD) [1], to improve both sensitivity and selectivity [2], to immobilize a larger quantity of bioreceptors [3] or even to change the transduction mechanism of the system [4]. Among them, noble metal nanostructures are emerging as functional materials in several fields thanks to their high stability and unique physicochemical properties [5].

A part from the effort to improve sensing performance using nanomaterials, lately there has also been a growing interest in the miniaturization of the devices for several applications. The recent wide spread of small point-of-care (POC) devices [6], the attempt to perform measurements in tiny spaces (like cells) [7] and the huge expansion of portable and wearable monitoring systems [8], have given a tremendous boost in this direction. In particular, in the last few years there has been a huge effort in the development of flexible non-invasive devices for a variety of applications [9]. The majority of the research works have focused their attention on non-invasive monitoring using alternative body fluids, like sweat, tears and saliva. These represents promising substitutes to blood analysis, thanks to their accessibility and non-invasiveness and to the possibility to reproduce them artificially in the lab [8,10]. Some remarkable advances have been made in wearable sweat sensors as this fluid is readily available and contains a huge amount of physiological information [11,12]. A discrete number of examples of complete miniaturized and wearable systems able to measures different electrolytes and metabolites can now be found in literature: these include sensors for the detection of glucose, lactate, ethanol and several ions [13,14,15,16,17,18,19]. Miniaturized ion-sensors find applications also in other expanding fields of application, that is on-line water quality monitoring. The development of small, cheap and sensitive on-line sensors that can be installed across distribution networks has attracted attention to improve water quality and reduce the risk of contamination [20]. However, sensor stability significantly lowers during prolonged storage in wet conditions [4,21,22,23].

In this paper we focus on the detection of lithium ions, while the technology can easily be extended to the monitoring of other ions. Lithium salts are still the most used mood stabilizers in psychiatric therapies. However, these drugs have a very narrow therapeutic range, thus the blood concentration needs to be controlled frequently to optimize the dose. Recently, a non-invasive decentralized method for monitoring of lithium drug concentration through sweat analysis was proposed [12].

The use of nanostructures has been found to be extremely beneficial in ion-sensing. In particular, they have been widely exploited as solid-contact (SC) in all-solid-state ion-selective sensors (ISE) to improve stability and reduce their inherent potential drift. SC-ISEs are fabricated by depositing an ion-selective membrane onto the solid substrate to selectively attract the ions in solution. The favorable impact of the nanostructures on sensor stability is due to the formation of an asymmetric capacitor as ions accumulate on the outer side of the ISM and electrons or holes are attracted on the inner side. The interfacial potential is thus proportional to the amount of charge in the electrical double layer, in contrast with respect to SCs based on conductive-polymers (CPs), where the potential depends on the redox reactions occurring in the material. Nanostructured-based SCs show some important advantages over CPs, including their hydrophobicity, large surface area, light insensitivity and high contact capacitance and the absence of side-reactions [4,24,25]. Both carbon and noble metals nanostructures have been used in SC-ISEs: they include carbon nanotubes [26,27,28,29], fullerene [30,31], graphene [29,32,33,34,35], polymer/carbon composites [36,37], porous carbon [38,39,40], gold nanoclusters [41], gold nanodendrites [24], nanoporous gold films [42], gold [43,44] and platinum [45] nanoparticles, platinum nanoflowers [46], combined platinum and gold nanostructures [47].

The use of noble metal nanostructures offers several advantages with respect to carbon-based materials, like their high stability, the non-toxicity and the possibility to use fast and conformal electrodeposition techniques for their fabrication [46,47,48]. In particular, we have recently proved that platinum nanoflowers deposited by a fast constant potential electrodeposition procedure on macro-electrodes allow the achievement of ISEs with high detection capabilities and exceptional stability. However, the used constant potential deposition is not effective on miniaturized micro-fabricated electrodes. In this paper, we investigate and optimize, for the first time, an alternative route to obtain platinum nanoflowers on micro-electrodes by means of linear sweep voltammetry (LSV). The electrodes were fabricated at the Holst centre—IMEC (The Netherlands), while their nanostructuration and characterization was performed at École Polytechnique Fédérale de Lausanne (EPFL), Switzerland. The effective deposition of platinum nanoflowers with similar features to the corresponding ones on macro-electrodes is confirmed by morphological analysis and by the similar roughness factor (~200) and capacitance values (~18 μF/mm${}^{2}$). Their electrochemical properties are then tested in a SC $Li^{+}$ micro-ISE and compared to the case of macro-electrodes. The fabricated miniaturized sensor offers Nernstian behaviour with same response time (~15 s) and a one-order of magnitude smaller LOD ($2.6\times 10^{-6}$) with respect to the macro-ISEs. Finally, the great reversibility and improved time-stability in both wet and dry conditions was proven.

## 2. Materials and Methods

### 2.1. Material

All chemicals were purchased from Sigma Aldrich (St. Louis, MO, USA).

### 2.2. Fabrication of Micro-Electrodes

Gold micro-electrodes were used for the deposition of the platinum nanoflowers. The gold electrodes were sputter deposited and patterned on an Si substrate, which was covered with a thermal ${\mathrm{SiO}}_{2}$ layer. Subsequently, a plasma-enhanced chemical vapor deposited (PECVD) ${\mathrm{SiO}}_{2}$ passivation layer was deposited and patterned on the gold electrodes using contact lithography leaving only the bond-pads and electrode area for the platinum deposition open. Finally, the wafer was diced and a single die with the gold electrodes was mounted and wire-bonded to a printed circuit board (PCB) with connectors. All bond-pads, wire-bonds and connector pads were covered by epoxy (Epotek H70e-2) to shield them from the fluids during the platinum deposition and the final use of the electrodes. The electrodes had a circular shape with a radius of 305 μm.

Platinum nanostructures were deposited by linear sweep voltammetry (LSV) in a 50 mM $H_{2}{\mathrm{SO}}_{4}$, 25 mM $H_{2}{\mathrm{PtCl}}_{6}$ aqueous solution using an Autolab PGSTAT 302N potentiostat with Nova software. A three-electrodes setup was employed with a Ag/AgCl double junction as a reference electrode (RE). Two different potential ranges were used: between 0 and –0.6 V for the procedures LSV1, between 0 and –0.8 V for LSV2.

The ion-selective membrane (ISM) was obtained by drop-casting 5 μL of a THF solution (1 wt % (6,6–dibenzyl–1,4,8–11–tetraoxacyclotetradecane), 28.00% poly (vinyl chloride) high molecular weight, 70.3 wt % 2–nitrophenyl octyl ether and 0.7 wt %, potassium tetrakis (4–chlorophenyl)borate)) onto the microfabricated electrodes. The solvent was allowed to evaporate overnight. The ion-selective electrodes (ISEs) were conditioned in $10^{-2}$ M LiCl for 1 day unless otherwise stated.

### 2.3. Morphological Characterization

The morphology of the samples was characterized by scanning electron microscopy (SEM). A thin iridium layer was deposited on the samples by evaporation in order to reduce surface charging due to the glass cover. The SEM analysis was performed either with a Merlin or a Gemini 300 microscope from Zeiss at the Interdisciplinary Centre of Electronic Microscopy (CIME) of EPFL in SE mode.

### 2.4. Electrochemical Characterization

Potentiometry was performed in a two-electrode setup using an EMF6 precision electrode interface by Lawson lab. Cyclic voltammetry and current reversal chronopotentiometry analysis were obtained with an Autolab potentiostat controlled by Nova Software in a three- and two-electrode configuration, respectively.

An Ag/AgCl double junction RE was used in all measurements with 3 M KCl as an internal electrolyte and a 1 M lithium acetate salt bridge.

## 3. Results and Discussion

### 3.1. Morphological Characterization

**Figure 1:**
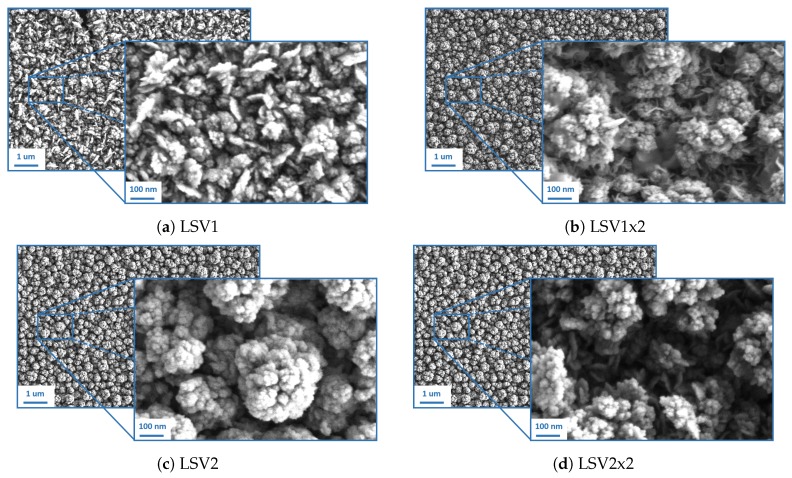
Scanning electron microscopy (SEM) images of platinum nanostructures deposited by linear sweep voltammetry (LSV) with different voltage ranges: between 0 and −0.6 V for the procedures called LSV1 (**a**), between 0 and −0.8 V for LSV2 (**c**). The comparisons with the structures obtained with two subsequent depositions is given in (**b**) and (**d**).

Two different LSV procedures have been investigated in this work to achieve a fast electrodeposition procedure of platinum nanostructures on miniaturized evaporated electrodes. In particular, two potential windows have been used: between 0 and −0.6 V for the procedure LSV1, between 0 and −0.8 V for LSV2. In addition, we have tested the difference in morphology when two subsequent identical deposition methods are applied on the same electrode. The SEM images of all resulting electrodes are shown in Figure 1. It is possible to notice that in the case of LSV1, a smaller quantity of platinum is deposited with respect to the others (Figure 1a). In fact, as a lower amount of material is transferred on the substrate, the architecture of platinum nanostructures appear to be simpler and less developed, although the nanofeatures are already present. As the potential window was extended (Figure 1c) or a double deposition was performed (Figure 1b) or both (Figure 1d), a higher amount of platinum was deposited on the electrode and more complex nanoflower-shaped structures were formed. No big variations in morphology were evident among the three nanostructures. These results were confirmed by comparing the roughness factors of the different platinum architectures, obtained as the ratio between the electrochemical active area (calculated from the area of the platinum oxide reduction peak in CV in sulphuric acid as described in [46]) and the geometrical area of the electrodes. Apart from LSV1 which shows a roughness factor of about 102.9 ± 1.7, all other nanostructures obtained by LSV attained very similar values (198.4 ± 0.3 for LSV1x2, 205.9 ± 0.1 for LSV2, 208.4 ± 1.5 for LSV2x2) to the one obtained on macro-electrodes by constant potential electrodeposition (201.8 ± 0.7). Thus, we can conclude that, despite the slight differences in morphology, the surface area of the different nanostructured layers are comparable. Consequently, only the faster single-step deposition methods will be further used for the electrochemical characterization of the SC electrodes since the more complex ones do not offer enough advantages.

It is also important to notice that the reproducibility of all nanostructures was very good, as proved by the standard deviation values of the roughness factors (0.3 for LSV1x2, 0.1 for LSV2, 1.5 for LSV2x2).

The following reactions can be involved in the platinum layer formation:

$$\mathrm{Pt}{\mathrm{Cl}}_{6}^{2-}+4e^{-}\to \mathrm{Pt}(s)+6{\mathrm{Cl}}^{-}$$

or

$$\mathrm{Pt}{\mathrm{Cl}}_{6}^{2-}+2e^{-}\to \mathrm{Pt}{\mathrm{Cl}}_{4}^{2-}+2{\mathrm{Cl}}^{-}$$

$$\mathrm{Pt}{\mathrm{Cl}}_{4}^{2-}+2e^{-}\to \mathrm{Pt}(s)+4{\mathrm{Cl}}^{-}$$

### 3.2. Current Reversal Chronopotentiometry (CRC) and SC Capacitance

**Figure 2:**
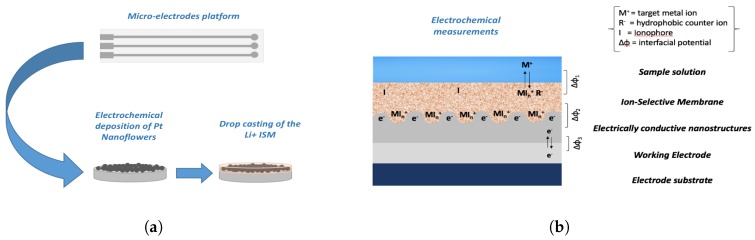
(**a**) A schematic illustration of the fabrication of the Li${}^{+}$ ion-selective sensors (ISE) based on the one-step electrodeposition of platinum nanostructures on micro-electrodes. The working mechanism is highlighted in (**b**).

The electrochemical behaviour of the platinum nanostructures fabricated on micro-electrodes were tested in a Li${}^{+}$ ISEs. The schematic illustration of the device fabrication and the working mechanism is given in Figure 2. Current reversal chronopotentiometry (CRC) measurements were performed to characterize the electrochemical performance of Li${}^{+}$ ISEs with and without nanostructured SCs. This technique represents a very useful way to determine the electrode capacitance and study the stability of the electrode. The sensor potential was measured during the application of a direct current of a few nA, that is then reversed. The typical E–t curve shows two main features: a jump as the current is reversed and a slow potential drift at longer times [49].

**Figure 3:**
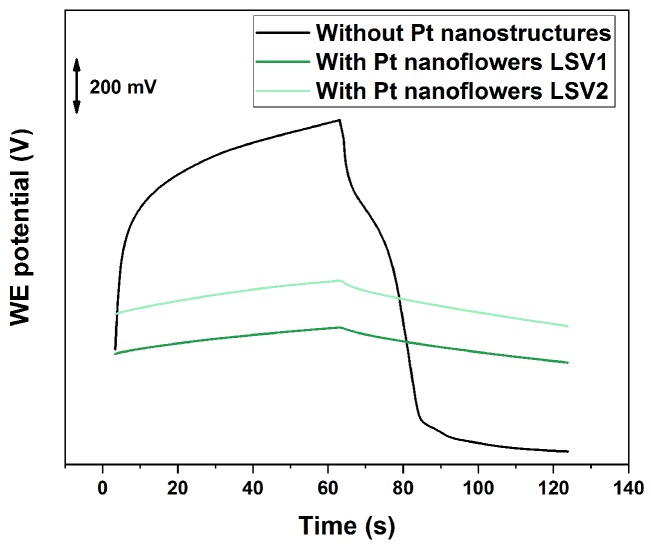
Current reversal chronopotentiometry (CRC) measurements of the different fabricated Li${}^{+}$ ISEs on micro-electrodes: without platinum nanostructures and with platinum nanoflowers deposited by LSV1 and LSV2.

The obtained curves are reported in Figure 3 in comparison with the electrodes without nanostructured SC. It is possible to see that the potential drift is significantly lowered when the surface area of the electrode is increased. This is a consequence of the different ion-to-electron transduction mechanism exploited by nanostructured materials and of their hydrophobic behaviour, which reduces the risk of water layer formation [4].

The SCs capacitance of the different electrodes can be calculated using to the following equation: $C=\frac{i}{dE/dt}$, where i is the applied current [4] and E the measured potential. The obtained values are reported in Table 1.

If we normalize the calculated capacitance by the area of the electrode, it is possible to notice that the obtained values are very close to the ones reported in [48] for similar structures deposited on macro-electrodes. This result confirms that the LSV procedure proposed in this work allows the formation of platinum nanostructures on micro-electrodes, with comparable results to the ones obtained on macro-electrodes by constant potential deposition. So, we can conclude that although LSV1 and LSV2 have some differences in morphology, as shown in Figure 1, the SC capacitance (which defines the stability of the electrode) is very similar. This is the most crucial property in ion-sensing as potential drift is the main issue related to the use of ISEs, especially on miniaturized electrodes.

### 3.3. Lithium-ISE Calibration

**Figure 4:**
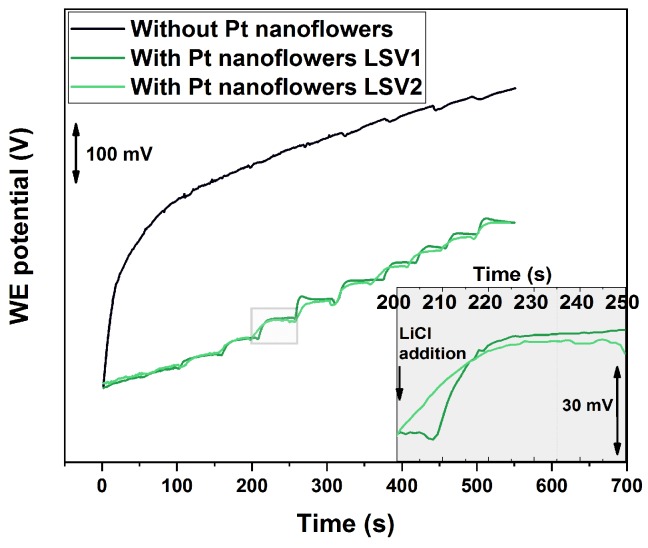
Calibration time traces of lithium-ISE fabricated on different solid-contact (SC). LiCl was added every 50 s to achieve a half-log increase of the concentration.

Lithium-ISE were fabricated on the different SCs and calibrated between $10^{-7}$ M and $10^{-1}$ M by subsequent additions of LiCl. The resulted time traces are reported in Figure 4. It is evident that the presence of platinum nanostructures significantly improves the sensor response. ISE without nanostructured SCs show enormous potential drift. As a consequence, the calibration steps are almost invisible. On the contrary, the curves obtained with platinum nanostructured-SCs (green lines) have clear and smooth features upon lithium addition.

**Figure 5:**
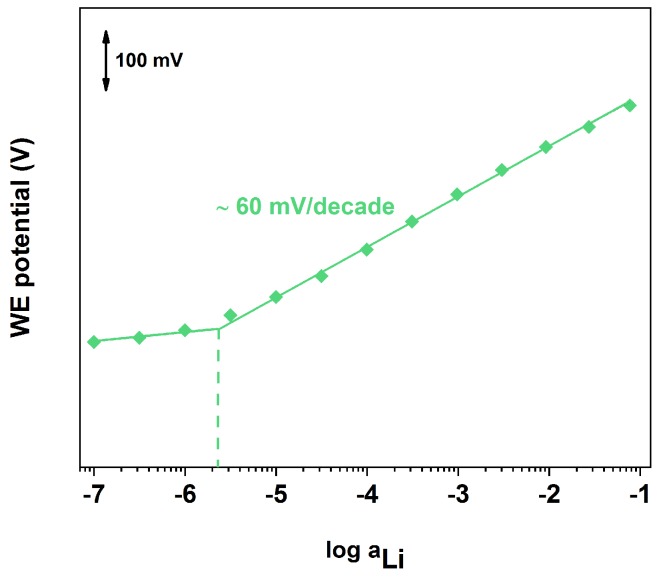
Calibration curve of lithium SC-ISE with platinum nanostructures deposited by LSV2.

From the potentiometric time traces given in Figure 4 it is possible to calibrate the sensors. An example is given in Figure 5 for the SC with platinum nanostructures deposited by LSV2. The sensor parameters obtained for all different micro-electrodes is given in Table 2 in comparison with the values reported in literature for macro-electrodes. From these results it is possible to conclude that all sensors offer Nernstian behaviour and short response time. In addition, the miniaturization of the electrodes reduced the LOD by half a order of magnitude with respect to the macro-electrodes in the case of LSV1 deposition. In the case of LSV2 deposition, the improvement in the LOD was even higher (one order of magnitude), while the standard deviation was significantly reduced (almost a half-order of magnitude). This can be explained by considering the higher surface area of these nanostructures, as discussed previously.

The membrane selectivity have already been investigated in [48]. It was proved to be very similar to the values of liquid junction ISEs.

### 3.4. Reversibility and Lifetime Studies

**Figure 6:**
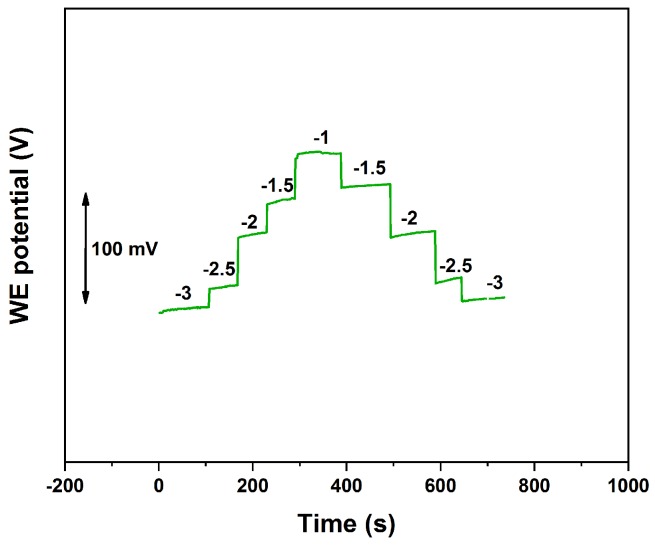
Reversed calibration between $10^{-3}$ and $10^{-1}$ M of for a Li${}^{+}$ SC-ISE on micro-electrodes. Platinum nanoflowers were deposited by LSV2.

Sensors reversibility was proved by performing a forward and backward calibration between $10^{-3}$ and $10^{-1}$ M, which is the range of interest in clinical applications for sweat analysis. A typical response for a SC-ISE obtained by LSV2 deposition of platinum nanostructures is given in Figure 6. It is evident that the device offers a stable and reversible response in the detection range.

**Figure 7:**
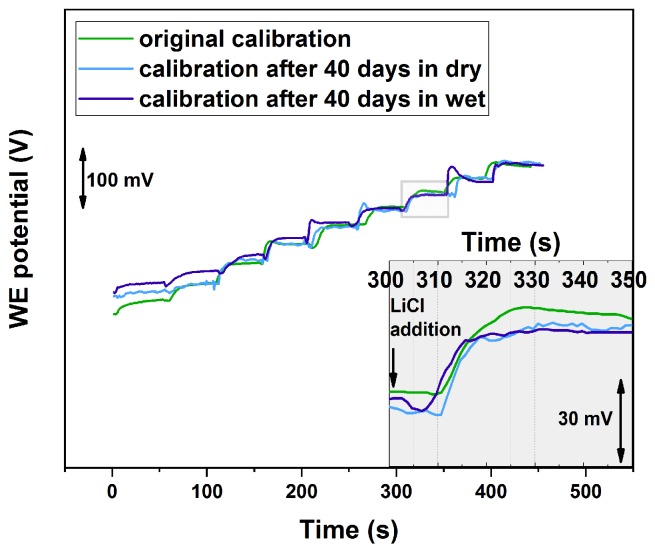
Calibration traces obtained from the freshly prepared Li${}^{+}$ SC-ISE (green) and the ones after 40 days of storage in dry (light blue) and then in wet (dark blue) conditions. Platinum nanoflowers were deposited by LSV2. LiCl solution was added every 50 s to achieve a half-log increase of the concentration.

Another important parameter in ion-sensing is the lifetime of the electrodes. In particular, it has been found in many articles [4,21,22,23] that the sensor performance significantly lowers when the sensor is kept in solution for a long period of time. Figure 7 shows the comparison among calibration traces obtained from the freshly prepared sample and the ones after 40 days of storage in dry or in wet conditions between $10^{-7}$ M and $10^{-1}$ M. A $10^{-2}$ M LiCl was used for wet storage. It is possible to notice that the sensor response remains almost the same, with smooth and sharp steps and Nernstian response in all cases (Table 3). Also, after 40 days of storage in solution, the sensor slopes and LOD decreased by less then 5%. This property is crucial in on-line applications where continuous exposure to wet conditions is needed.

## 4. Conclusions

Platinum nanostructures have been found to have exceptional properties in sensing applications, especially for ion-selective electrodes. However, the classical deposition by applying constant potential is not suitable for miniaturized evaporated electrodes. In this paper, we developed a simple and efficient electrodeposition procedure for miniaturized electrodes based on LSV. Two potential windows and successive depositions are investigated and compared. Single-step procedures produce similar morphological features while allowing a simpler fabrication protocol. Although the two LSV depositions produce differently shaped platinum nanoflowers, when used to fabricated a SC-ISE, their electrochemical behaviour is very similar. The capacitance values of the two structures obtained by CRC measurements is almost equal. Furthermore, the value is very close to the one reported in literature for platinum nanostructures on macro-electrodes, which is a highly notable result. All sensors show short response time (∼15 s) and Nernstian calibration with one-order of magnitude lower detection limit ($2.6\times 10^{-6}$) with respect to the micro-electrodes values. Finally, sensor reversibility and stability both in wet and dry conditions are also confirmed.

## Figures and Tables

**Table 1:** Potential drift and capacitance values obtained from the Current reversal chronopotentiometry (CRC) measurements of the different fabricated Li${}^{+}$ ion-selective sensors (ISE) on micro-electrodes in comparison with the literature values on macro-electrodes.

	dE/dt	C	Normalized C
	[mV/s]	[μF]	[μF/mm${}^{2}$]
Without Pt Nanostructures (micro-electrodes)	1.02 ± 0.22	0.57 ± 0.17	4.35 ± 1.29
LSV1 (micro-electrodes)	0.23 ± 0.08	2.55 ± 0.41	19.39 ± 1.29
LSV2 (micro-electrodes)	0.18 ± 0.01	2.39 ± 0.19	18.15 ± 3.09
Literature (macro-electrodes) [48]	–	–	15.55 ± 7.71

**Table 2:** Sensor parameters obtained from the calibration curves of the different fabricated Li${}^{+}$ ISEs on micro-electrodes in comparison with the literature values on macro-electrodes. (Calibration range between $10^{-7}$ M and $10^{-1}$ M.)

	Slope [mV/decade]	LOD	Response Time [s]
LSV1 (micro-electrodes)	61.7 ± 3.7	$\left(4.4\pm 3.9\right)\times 10^{-6}$	15–30
LSV2 (micro-electrodes)	59.0 ± 1.0	$\left(2.6\pm 0.5\right)\times 10^{-6}$	15–30
Literature (macro-electrodes) [48]	58.7 ± 0.8	$\left(13.0\pm 4.0\right)\times 10^{-6}$	15–30

**Table 3:** Sensor parameters obtained from the calibration traces in Figure 7 for the freshly prepared Li${}^{+}$ SC-ISE and the ones after 40 days of storage in dry and then in wet conditions. (Calibration range between $10^{-7}$ M and $10^{-1}$ M.)

	Slope [mV/decade]	LOD	Response Time [s]
As prepared	59.1	$2.7\times 10^{-6}$	~15 s
After 40 days in dry	59.0	$2.7\times 10^{-6}$	15–20
After 40 days in wet	56.5	$2.8\times 10^{-6}$	15–40

## Abbreviations

The following abbreviations are used in this manuscript:

CRC	current reversal chronopotentiometry
CP	conductive polymer
CV	cyclic voltammetry
ISE	ion-selective electrode
ISM	ion-selective membrane
LOD	limit of detection
LSV	linear sweep voltammetry
POC	point-of-care
RE	reference electrode
SC	solid contact
SEM	scanning electron microscopy
WE	working electrode
